# Impact of systematic early tuberculosis detection using Xpert MTB/RIF Ultra in children with severe pneumonia in high tuberculosis burden countries (TB-Speed pneumonia): a stepped wedge cluster randomized trial

**DOI:** 10.1186/s12887-021-02576-5

**Published:** 2021-03-20

**Authors:** Aurélia Vessière, Hélène Font, Delphine Gabillard, Laurence Adonis-Koffi, Laurence Borand, Chishala Chabala, Celso Khosa, Sandra Mavale, Raoul Moh, Veronica Mulenga, Juliet Mwanga-Amumpere, Jean-Voisin Taguebue, Mao Tan Eang, Christophe Delacourt, James A. Seddon, Manon Lounnas, Sylvain Godreuil, Eric Wobudeya, Maryline Bonnet, Olivier Marcy

**Affiliations:** 1 University of Bordeaux, Inserm, Institut de Recherche pour le Développement (IRD), UMR 1219, Bordeaux, France; 2 Paediatrics Department, Yopougon University Hospital, Abidjan, Ivory Coast; 3 Epidemiology and Public Health Unit, Pasteur Institute in Cambodia, Phnom Penh, Cambodia; 4 University of Zambia School of Medicine, University Teaching Hospital, Lusaka, Zambia; 5 Instituto Nacional de Saúde, Maputo, Mozambique; 6 Paediatrics Department, Maputo Central Hospital, Maputo, Mozambique; 7 Programme PAC-CI, CHU de Treichville, Abidjan, Ivory Coast; 8 Children’s Hospital, University Teaching Hospital, Lusaka, Zambia; 9 Epicentre Mbarara Research Centre, Mbarara, Uganda; 10 Mother and Child Center, Chantal Biya Foundation, Yaoundé, Cameroon; 11 National Center for Tuberculosis and Leprosy (CENAT/NTP), Ministry of Health, Phnom Penh, Cambodia; 12 Department of Paediatric Pulmonology, Necker University Teaching Hospital, Paris, France; 13 Desmond Tutu TB Centre, Department of Paediatrics and Child Health, Stellenbosch University, Cape Town, South Africa; 14 Department of Infectious Diseases, Imperial College London, London, UK; 15 IRD-Mivegec/CNRS U5290, Montpellier, France; 16 Makerere University-Johns Hopkins University Research Collaboration (MU-JHU) Care Ltd, Kampala, Uganda; 17 IRD UMI233/Inserm U1175, University of Montpellier, Montpellier, France

**Keywords:** Children, Pneumonia, Tuberculosis, Nasopharyngeal aspirate, Stool, Xpert MTB/RIF ultra

## Abstract

**Background:**

In high tuberculosis (TB) burden settings, there is growing evidence that TB is common in children with pneumonia, the leading cause of death in children under 5 years worldwide. The current WHO standard of care (SOC) for young children with pneumonia considers a diagnosis of TB only if the child has a history of prolonged symptoms or fails to respond to antibiotic treatments. As a result, many children with TB-associated severe pneumonia are currently missed or diagnosed too late. We therefore propose a diagnostic trial to assess the impact on mortality of adding the systematic early detection of TB using Xpert MTB/RIF Ultra (Ultra) performed on nasopharyngeal aspirates (NPA) and stool samples to the WHO SOC for children with severe pneumonia, followed by immediate initiation of anti-TB treatment in children testing positive on any of the samples.

**Methods:**

TB-Speed Pneumonia is a pragmatic stepped-wedge cluster randomized controlled trial conducted in six countries with high TB incidence rate (Côte d’Ivoire, Cameroon, Uganda, Mozambique, Zambia and Cambodia). We will enrol 3780 children under 5 years presenting with WHO-defined severe pneumonia across 15 hospitals over 18 months. All hospitals will start managing children using the WHO SOC for severe pneumonia; one hospital will be randomly selected to switch to the intervention every 5 weeks. The intervention consists of the WHO SOC plus rapid TB detection on the day of admission using Ultra performed on 1 nasopharyngeal aspirate and 1 stool sample. All children will be followed for 3 months, with systematic trial visits at day 3, discharge, 2 weeks post-discharge, and week 12. The primary endpoint is all-cause mortality 12 weeks after inclusion. Qualitative and health economic evaluations are embedded in the trial.

**Discussion:**

In addition to testing the main hypothesis that molecular detection and early treatment will reduce TB mortality in children, the strength of such pragmatic research is that it provides some evidence regarding the feasibility of the intervention as part of routine care. Should this intervention be successful, safe and well tolerated, it could be systematically implemented at district hospital level where children with severe pneumonia are referred.

**Trial registration:**

ClinicalTrials.gov, NCT03831906. Registered 6 February 2019.

## Background

The World Health Organization (WHO) estimated that 1.12 million children (< 15 years) developed tuberculosis (TB) in 2018, representing 11% of the overall TB case load. WHO also estimated 205,000 child TB deaths that year [1], making TB a top ten cause of death in children under five years worldwide [2]. Recent modelling suggests that almost all children dying from TB (96%) are untreated and that 80% are aged below five years, with treatment not started likely due to not being diagnosed with TB [2]. Indeed, only 512,000 paediatric TB cases were notified to WHO in 2018, representing a treatment coverage of approximately 46% [1]. Childhood TB therefore remains undiagnosed and underreported, mostly due to the challenges in confirming its diagnosis. This in turn is largely because of the paucibacillary nature of disease, and the difficulty in obtaining respiratory samples from young children [3].

Pneumonia is the leading cause of death in children under the age of five years worldwide. There were an estimated 120 million pneumonia episodes in children younger than 5 years in 2011, including 14 million severe episodes [4], of which 1.3 million led to death. In 2014, WHO revised its definitions for pneumonia and severe pneumonia, the latter only justifying referral from primary health centres to higher level facilities for inpatient treatment [5]. However, there are no global estimates of mortality specifically attributable to severe pneumonia as currently defined by WHO. Studies in children hospitalized with severe pneumonia conducted in East and East-central Africa showed an inpatient mortality ranging from 8.7 to 32% [6–8]. Children yet remain at high risk for death even after discharge, notably during the first 3 months, with severe acute malnutrition an important factor of poor outcome [6].

In high TB burden settings, there is growing evidence that TB is common in children with pneumonia [9]. Although TB is a chronic disease in adults, recent data shows that the duration of respiratory symptoms before admission can be acute in children with severe pneumonia associated with TB [10]. A systematic review showed that up to 23% of children admitted to hospital with an initial diagnosis of pneumonia were later diagnosed with TB [10, 11]. This is particularly true in the African and South-East Asian WHO regions, which accounted for 30 and 40% of all paediatric TB cases in 2019 respectively [1]. In these regions, the case fatality rate for childhood pneumonia associated with TB is high, ranging from 4 to 21% [10], with younger age, malnutrition and HIV infection increasing the risk of death [12, 13]. However, the diagnosis of TB in children with severe pneumonia remains low. Indeed, the current WHO standard of care (SOC) for young children with pneumonia considers a diagnosis of TB only if the child has a history of prolonged symptoms or fails to respond to antibiotics [14]. Therefore, many children with TB-associated severe pneumonia are currently missed or diagnosed too late, which is likely to affect their outcome.

In 2013, WHO updated its policy to include Xpert MTB/RIF (Cepheid, USA) as the initial test for the diagnosis of TB in children, based on a meta-analysis showing a pooled sensitivity and specificity of Xpert MTB/RIF performed on gastric lavages of 66% (95% confidence interval 51–81) and 98% (95% confidence interval 96–99), respectively, when compared with culture [15, 16]. Although data on the performance of Xpert MTB/RIF in children with pneumonia are limited, in Bangladesh, sensitivity on gastric aspirates or sputum samples compared to culture in this group was equivalent to that reported in other studies [17]. The next-generation of Xpert MTB/RIF assay, Xpert MTB/RIF Ultra (Ultra), has a lower detection threshold (similar to culture), which is expected to improve the diagnosis of children with paucibacillary TB [18, 19].

Previous studies in Africa and Asia have shown that alternative specimen collection methods such as nasopharyngeal aspirates (NPA) and stool samples are easier to implement, and are better tolerated in young and sick children [20–24]. These methods do not require a child to fast, as mandatory for gastric aspirates, and are more suitable than induced sputum in children with severe respiratory deficits [25]. Recent studies have shown similar sensitivity of Xpert MTB/RIF on the combination of one stool and one NPA as compared to two induced sputum or two gastric aspirates [20, 26].

We therefore propose a diagnostic trial to assess the impact on mortality of adding the systematic early detection of TB using Ultra performed on NPA and stool samples to the WHO SOC for children with severe pneumonia, followed by immediate initiation of anti-TB treatment in children testing positive on any of the samples. Our hypothesis is that in high TB burden countries, testing young children with severe pneumonia for TB and starting those who test positive on anti-TB treatment on the day of presentation, could reduce all-cause mortality through reduction of mortality attributed to TB.

## Methods

### Aim

The primary objective of the TB-Speed Pneumonia trial is to evaluate the impact on all-cause mortality at 12 weeks post inclusion of adding systematic early detection of TB with Ultra, performed on one NPA and one stool sample in young children with severe pneumonia, followed by immediate anti-TB treatment initiation in children with a positive Ultra result, in high TB incidence countries, as compared to the WHO SOC alone. The study only includes children with community-acquired pneumonia, excluding children already hospitalized who have developed a nosocomial pneumonia.

Secondary objectives will assess the impact of the systematic TB detection on TB case detection, time to TB treatment initiation, inpatient mortality, duration of initial hospitalization and hospital readmission rate, and will assess the feasibility and acceptability of the intervention.

### Trial design

TB-Speed Pneumonia is an international, cluster-randomised trial with a stepped wedge design. Stepped wedge trials are randomised controlled trial in which clusters successively switch from control to intervention, in an order randomly assigned, until all clusters are eventually exposed to the intervention (Table 1). In our study, all hospitals (clusters) start by implementing the WHO SOC for severe pneumonia (control arm) and are randomly allocated a time at which they will transition to implementing the intervention. Depending on the time at which they are enrolled in the study, children pertain either - and exclusively – to the control arm, or the TB-Speed intervention arm.

**Table 1:** Stepped wedge implementation of the intervention in participating hospitals

TB incidence rate	Hospital number	Period (5-week intervals)															
		01	02	03	04	05	06	07	08	09	10	11	12	13	14	15	16
High	01	CT	INT	INT	INT	INT	INT	INT	INT	INT	INT	INT	INT	INT	INT	INT	INT
**Very high**	**02**	CT	CT	INT	INT	INT	INT	INT	INT	INT	INT	INT	INT	INT	INT	INT	INT
High	03	CT	CT	CT	INT	INT	INT	INT	INT	INT	INT	INT	INT	INT	INT	INT	INT
**Very high**	**04**	CT	CT	CT	CT	INT	INT	INT	INT	INT	INT	INT	INT	INT	INT	INT	INT
High	05	CT	CT	CT	CT	CT	INT	INT	INT	INT	INT	INT	INT	INT	INT	INT	INT
**Very high**	**06**	CT	CT	CT	CT	CT	CT	INT	INT	INT	INT	INT	INT	INT	INT	INT	INT
High	07	CT	CT	CT	CT	CT	CT	CT	INT	INT	INT	INT	INT	INT	INT	INT	INT
**Very high**	**08**	CT	CT	CT	CT	CT	CT	CT	CT	INT	INT	INT	INT	INT	INT	INT	INT
High	09	CT	CT	CT	CT	CT	CT	CT	CT	CT	INT	INT	INT	INT	INT	INT	INT
**Very high**	**10**	CT	CT	CT	CT	CT	CT	CT	CT	CT	CT	INT	INT	INT	INT	INT	INT
High	11	CT	CT	CT	CT	CT	CT	CT	CT	CT	CT	CT	INT	INT	INT	INT	INT
**Very high**	**12**	CT	CT	CT	CT	CT	CT	CT	CT	CT	CT	CT	CT	INT	INT	INT	INT
High	13	CT	CT	CT	CT	CT	CT	CT	CT	CT	CT	CT	CT	CT	INT	INT	INT
**Very high**	**14**	CT	CT	CT	CT	CT	CT	CT	CT	CT	CT	CT	CT	CT	CT	INT	INT
High	15	CT	CT	CT	CT	CT	CT	CT	CT	CT	CT	CT	CT	CT	CT	CT	INT

*CT* control (WHO recommended standard of care for children with severe pneumonia), *INT* TB-Speed intervention (systematic early detection of tuberculosis in addition to the WHO recommended standard of care for children with severe pneumo*nia*)

### Study settings

The impact of this innovative approach may vary with TB incidence as well as geographical and seasonal variability that can affect the prevalence and aetiology of pneumonia in young children. To provide a better basis for the generalisability of results, the trial takes place in six countries. These include high and very high tuberculosis incidence countries with different epidemiological and environmental backgrounds, in Sub-Saharan Africa (Cameroon, Cote d’Ivoire, Mozambique, Uganda, and Zambia) and South East Asia (Cambodia) (Table 2). The trial is implemented in 15 national or regional reference hospitals with previous research experience.

**Table 2:** Implementing sites

Region	Country	TB incidence rate /100,000 population		Number of sites	Hospitals, City
Western Africa	Côte d’Ivoire	High (< 300)	148	3	Yopougon UTH, Abidjan
					Treichville UTH, Abidjan
					Cocody UTH, Abidjan
Central Africa	Cameroon		194	2	Chantal Biya Foundation, Yaoundé
					District Hospital Biyem Assi, Yaoundé
Eastern Africa	Uganda		201	3	Mulago National Referral Hospital, Kampala
					Holy Innocents Childrens’ Hospital, Mbarara
					Regional Reference Hospital, Jinja
Southern Africa	Mozambique	Very high (≥300)	551	2	Central Hospital, Maputo
					Jose Macamo General Hospital, Maputo
	Zambia		361	2	UTH, Lusaka
					Arthur Davidson Children Hospital, Ndola
South East Asia	Cambodia		326	3	Referral Hospital, Kampong Cham
					Referral Hospital, Takeo
					National Pediatric Hospital, Phnom Penh

*UTH* University Teaching Hospital. Data source: WHO Global TB Report 2019 [1]

### Randomisation

Randomisation is stratified by the estimated country TB incidence rate, classified as either high (100 to < 300/100,000 patients-years; Cameroon, Côte d’Ivoire and Uganda) or very high (≥ 300/100,000 patients-years; Cambodia, Mozambique and Zambia) [9] (Table 1). Within these strata, a computer-generated random sequence will determine the order in which hospitals move from control to intervention. The statistician of the international coordination team, based at University of Bordeaux, will prepare the randomization sequence before the start of the trial.

The time when a new cluster receives the intervention is called a step. In the TB-Speed Pneumonia study, the period between two successive steps is 5 weeks (Table 1). The international coordination team and country research teams are blinded to the randomisation order. The international coordination team however is informed 10 weeks in advance (i.e. two periods) of the next site to switch, while study sites are notified 5 weeks prior to their crossover date to initiate program planning.

### Study population

Any child younger than five years presenting with signs and symptoms of presumptive severe pneumonia to outpatient, emergency units, intensive care unit, or paediatric departments of the selected hospitals is screened for eligibility for the trial as soon as possible. Eligibility to participate includes the following criteria: (1) aged 2 to 59 months, (2) newly hospitalized for WHO-defined severe pneumonia (Table 3), (3) informed consent signed by parent/guardian. Ongoing TB treatment or history of intake of anti-TB drugs in the last 6 months is the only exclusion criterion.

**Table 3:** WHO criteria for severe pneumonia

MANDATORY	PLUS ≥ 1 of the following	
**Cough OR difficulty in breathing**	Peripheral oxygen saturation < 90%	
	Central cyanosis	
	Severe respiratory distress	Grunting
		Nasal flaring
		Very severe chest indrawing
	Signs of pneumonia (tachypnea OR chest indrawing) AND at least one danger signs (a to f)	(a) Inability to breastfeed or drink
		(b) Persistent vomiting
		(c) Lethargy or reduced level of consciousness
		(d) Convulsions
		(e) Stridor in calm child
		(f) Severe malnutrition

### Trial intervention strategy

#### The WHO standard of care for children with severe pneumonia

All children admitted in the hospital and presenting with WHO-defined severe pneumonia are immediately managed as part of routine care per the WHO SOC for children with severe pneumonia (Table 4).

**Table 4:** The WHO standard of care for young children with severe pneumonia

Care	Conditions
Antibiotics	Broad spectrum intravenous antibiotics
Oxygen therapy	If oxygen saturation < 90% or signs of hypoxia
Additional supportive care	Airway management, fever treatment, bronchodilators or steroids, fluids and nutritional support (including breastfeeding or nasogastric tube if needed)
Specific therapies for comorbidities	HIV infection, malnutrition
Chest X-ray	If possible, for children with severe pneumonia not responding to treatment or complications or unclear diagnosis or associated with HIV
Monitoring	By a nurse at least every 3 h and by a doctor at least twice a day
Follow up	If possible, 2 weeks after discharge, to check the child’s nutrition

As recommended by the WHO for TB assessment in the context of the SOC, if the child presents with persistent cough and fever for more than two weeks, and signs of pneumonia after adequate antibiotic treatment, s/he is evaluated for TB using routine procedures. Xpert or Ultra, depending on local availability, can be used in children with clinical suspicion of TB (chronic symptoms, failure to respond to antibiotic treatment or TB exposure) according to the clinician’s judgement. This can be done using the standard sample collection methods usually implemented at the inpatient ward.

#### The TB-Speed intervention

The intervention consists of the WHO SOC for children with severe pneumonia plus the trial intervention consisting of systematic, rapid detection of TB on the day of hospital admission using the Ultra assay performed on 1 NPA and 1 stool sample. Ultra testing on NPA is performed immediately, either at the hospital laboratory with the standard GeneXpert device, or undertaken in the ward or in a side-laboratory next to the ward using a one-module GeneXpert device (G1 Edge®, Cepheid). The sample flow has been organised in order to reduce turnaround time for results to 3 h. Ultra testing on stool, which requires prior processing, is performed at the hospital laboratory. Drugs are available at the inpatient level to enable immediate initiation of TB treatment, as soon as a positive Ultra result is released.

### Implementation procedure

Aggregated data on severe pneumonia management were collected in all hospitals two months prior to the start of the study to document routine practices for the SOC for severe pneumonia in children (number of hospitalizations, inpatient mortality, antibiotic use, access to oxygen therapy and other supportive care). Adherence to the WHO SOC for severe pneumonia was reinforced by initial study training and is monitored throughout the study implementation. The study provided equipment for oxygen therapy (oxygen concentrators) where needed, and pulse oximeters. It is expected that the provision of equipment for oxygen therapy and training on pneumonia case management in children will reduce the variation in characteristics and practices between sites.

### Study visits and assessments

After written informed consent is obtained from parent(s)/guardian(s) by the study clinician or study nurse, the baseline visit includes a complete clinical evaluation, a digitalized chest X-ray (CXR), HIV and malaria testing, and a complete blood count. For children in the intervention arm, initial bacteriological specimen collection is done as soon as possible and within 24 h of hospital admission, including one NPA collected by the nurse on the day of admission, and one stool sample collected as soon as the child is able to produce stool. Additionally, blood samples and leftovers from NPA and stool are collected for future biomarkers studies in children for whom parent(s)/guardian(s) give their consent for biobanking. Data about routine care as well as additional study strategies are collected. All children are followed up for a total duration of 12 weeks, with four systematic protocol visits planned at day 3 (and/or at hospital discharge), 2 weeks after discharge (as recommended by the WHO SOC), and 12 weeks after recruitment. Each follow-up visit comprises a clinical evaluation, and collection of medical history since the last visit, an evaluation of adherence to TB treatment if initiated, and TB drug prescription and dispensation to cover the time until the next visit (Table 5). Parent(s)/guardian(s) are invited to bring their child back to the hospital in case of new symptoms for an unscheduled (extra) visit. During the final visit at 12 weeks, a second digital CXR is performed, as well as an assessment of TB disease evolution and TB treatment outcome in those diagnosed with TB (improvement, treatment failure, death, or lost to follow-up).

**Table 5:** Study assessments and specimen collection

	Inclusion (Day 0)	Day 3	Discharge	2 Weeks Post-Discharge	(Extra visit)^**i**^	Week 12
**STANDARD OF CARE (control and intervention arm)**						
WHO Standard of Care^a,b^	X	X	X	X	(X)	X
TB clinical assessment^c^		(X)	(X)	(X)	(X)	(X)
TB treatment if needed^b, d^		(X)	(X)	(X)	(X)	(X)
**TB-SPEED INTERVENTION (intervention arm only)**						
Nasopharyngeal aspirate	X					
Stool sample	X					
Xpert MTB/RIF Ultra	X					
Immediate TB treatment initiation if positive Ultra test^b^	X					
Biobank: NPA and stool leftovers	X					
Tolerability and acceptability of NPA collection^h^	X					
**ADDITIONAL STUDY ASSESSMENT AND PROCEDURES (control and intervention arm)**						
Eligibility screening	X					
Clinical evaluation^e^	X	X	X	X	(X)	X
Medical history	X	X	X	X	(X)	X
Digital chest X-Ray	X				(X)	X
Safety assessment	X	X	X	X	(X)	X
TB drug adherence assessment		(X)	(X)	(X)		(X)
TB treatment response				X		X
HIV test^f^	X					
Malaria test	X					
Complete blood count	X					
Biobank: plasma, whole blood^g^	X					

*WHO* World Health Organization, *TB* tuberculosis, *NPA* nasopharyngeal aspirate

^a^See Table 4

^b^According to national treatment guidelines based on WHO recommendations

^c^In children with a clinical suspicion of TB (chronic symptoms, failure to respond to antibiotic treatment or TB exposure), TB will be evaluated using routine procedures. This can include Xpert MTB/RIF or Ultra (depending on local availability) on standard bacteriological samples as usually implemented at the ward

^d^In the intervention arm, TB treatment will be initiated immediately in case of a positive Ultra result. In both arms, TB treatment could be initiated in case of a strong clinical suspicion

^e^Content of clinical evaluation varies with the visit; includes TB exposure and symptoms assessment at inclusion

^f^Performed if not available in the patient medical chart. In Côte d’Ivoire, should be discriminant for HIV 1 and 2

^g^In children < 18 months weighing < 5 kg, or presenting with signs of severe anaemia (conjunctival or palmar pallor): plasma sample for biobank will not be collected. Overall, volume of blood draw must not exceed 3 ml/kg/visit and 7 ml/kg/6 weeks

^h^In a subset of children only

^i^An extra TB visit will be performed if the child presents with signs and symptoms in favour of a presumptive TB

#### Tolerability and acceptability of the intervention

Assessment of the tolerability and acceptability of NPA and stool specimen collection procedures will be undertaken in a subset of children. Tolerability is defined by the child’s perceived level of discomfort/distress/pain as assessed by the child him/herself, the parents/guardians and the nurses using the Wong-Baker Face scale, the Visual Analog Scale, and the FLACC (Face Legs Activity Cry Consolability) behavioural scale, respectively. Nurses’ and parents’ acceptability regarding the whole sampling and testing strategy will be evaluated using both quantitative (self-reported questionnaire) and qualitative methods (semi-structured interviews).

#### Safety assessment

Occurrence of Adverse Events (AEs) is monitored at each visit by study nurses and clinicians for children receiving the TB-Speed intervention. Expected AEs occurring from NPA collection include, by decreasing order of frequency: cough, nausea, local trauma/nose bleeding, sneezing, vomiting, and in rare cases dyspnoea/low oxygen saturations and bradycardia < 60/bpm [33]. No AEs are expected from stool sample collection. Since this is a diagnostic trial without investigational medicinal product, and very low expected risk of AEs linked to the intervention, there is no systematic notification of severe AEs (SAEs) to the sponsor with the exception of: 1) death; 2) grade 4 clinical AEs (excluding asymptomatic biological grade 4 AEs); or 3) SAEs related to NPA collection. Grading will be done using the 2017 Division of AIDS Table for Grading the Severity of Adult and Pediatric Adverse Events [27].

### Endpoints

#### Primary study endpoint and measure

The primary endpoint is all-cause mortality 12 weeks after recruitment. Mortality due to severe pneumonia (related or not to TB) is expected to occur early. We expect that a 12-week period is long enough to assess impact on mortality of TB treatment empirically started in children with poor clinical progress during the first weeks of follow-up. If the children are not brought to the Week 12 visit, parents will be contacted, and home visits will be organized in order to collect the vital status of the child.

#### Secondary study endpoints and measures

Several secondary endpoints will further compare the intervention and control arms and document the feasibility and acceptability of the intervention (Table 6). Of note, some endpoints are collected all along the trial for process monitoring and assessment of the feasibility of the TB-Speed intervention. Among others, they include the *proportion of children with NPA and stool samples collected as per protocol* and the *time to sample collection and Xpert result after collection.*

**Table 6:** Secondary endpoints

Endpoints	Measures	Time of measurement
***Secondary endpoints considered for a comparison between arms***		
TB diagnosis based on the clinician’s judgement	# of children diagnosed with TB based on the clinician’s judgement	Any time during the follow-up
TB treatment initiation	Proportion of children diagnosed with TB AND who with at least one TB treatment recorded	Any time during the follow-up
Time to TB treatment initiation	Date/Time of the 1st TB treatment – Date/Time of TB diagnosis (in hours)	Any time during the follow-up
Duration of TB treatment at end of trial	Number of days between date of TB treatment initiation and date of TB treatment end	Any time during the follow-up (week 12 or early termination)
Inpatient deaths	# of children who died after inclusion and before hospital discharge	Before discharge
Duration of initial hospitalization	Date of the 1st recorded discharge – Date of the 1st recorded admission (in days)	At the first discharge
Readmission following discharge	# of admission occurring after the 1st discharge	Any time during the follow-up
Weight gain	Proportion of weight gain at 12 weeks (as compared to body weight at inclusion)	At 12 weeks
Cost effectiveness	Incremental cost-effectiveness ratio (ICER) of intervention compared with WHO SOC, measured in cost per DALY averted	Lifetime, based on differences in mortality up to 12 weeks
***Secondary endpoints assessed in the intervention group only***		
TB detection by NPA testing	Proportion of NPA with positive TB detection using Ultra	After 1st visit
TB detection by Stool testing	Proportion of stool samples with positive TB detection using Ultra	After 1st visit
TB detection by NPA and Stool testing	Proportion of samples (NPA and/or stool) with positive TB detection using Ultra	After 1st visit
Turnaround time between NPA collection and result of Ultra	Date/Time of Ultra results – Date/Time of NPA collection	After 1st visit
Turnaround time between stool sample collection and result of Ultra	Date/Time of Ultra results – Date/Time of stool sample collection	After 1st visit
NPA collected as per protocol	Proportion of children with NPA collected as per protocol	At 1st visit
Stool samples collected as per protocol	Proportion of children with stool samples collected as per protocol	At 1st visit
Safety: adverse events (AEs) during NPA collection	Number of adverse events collected by study nurses during NPA collection including as vomiting, nose bleeding, low oxygen saturation	Any time during the follow-up
Tolerability: discomfort/pain/distress experienced by the child during NPA collection procedure	Assessed by the child him/herself (Wong-Baker face scale), by the parents (visual analog scale), by the nurses (FLACC behavioural scale)	At NPA collection time

*TB* tuberculosis, *NPA* nasopharyngeal aspirate

### Data analysis

We will perform final data analysis to answer the objectives at the end of the study after data review and database closure. At the request of the trial Independent Data Monitoring Committee (IDMC), an interim analysis on safety and feasibility data is planned at the point when all children enrolled during the first 9 months of the study have been followed for 12 weeks; there are no stopping rules planned and no formal statistical test will be used. We will conduct a descriptive analysis of the endpoints and other measured variables and characteristics (Table 6). Quantitative variables will be summarized using means, standard deviation, median and interquartile range. Categorical variable will be analysed using frequencies and proportions.

The analysis of the primary endpoint will be performed following an intention-to-treat principle. Patterns of missing values will be analysed but no imputation will be done. As the literature regarding statistical analysis of stepped wedge designs is constantly growing, we will follow the recommendations of Hemming and colleagues [28], using the Hussey and Hughes model for the primary analysis [29]. Thus, we will used generalised linear mixed models with logit link and binary distribution. We will model individual binary response (vital status) with condition (control vs intervention) and time-period as fixed effects and sites as random effects to adjust for clustering of children. Sensitivity analyses will be performed to control for 1) important prognostic factors such as age, malnutrition, HIV infection, severity criteria measured at recruitment, 2) seasonality which could be different in different countries and, 3) different model specifications to better adjust secular trends and cluster heterogeneity [28]. These model specifications are developed and detailed in a statistical analysis plan that will be validated by the IDMC before performing the analysis. All results will be reported as estimates of effect (odds ratios for binary variables) and corresponding 95% confidence intervals. We will conclude that the intervention is better than the control if the mortality odds ratio is statistically lower than 1 (*p* < 0.05).

All analyses will be performed using R (version 3.6.0 or higher) and findings will be reported as per the CONSORT extension for reporting of stepped-wedge cluster-randomized trials [30].

### Power and sample size

Sample size calculations were performed based on several assumptions. (1) The expected proportion of TB cases in children with pneumonia would be 15 and 24%, in high and very high incidence settings, respectively, with a proportion of 33% of culture confirmed cases among TB cases overall (2). In the context of our intervention, Ultra could detect the majority of confirmed TB cases, versus a hypothesized TB detection rate of 25% in routine conditions. (3) The intervention would raise awareness about TB in site clinicians and would lead to an increase in detection rate from 15 to 50% between the control and the intervention arm. (4) We estimate that in the control arm the overall mortality will reach 15%, in line with mortality associated to severe pneumonia in previous studies, and that the intervention would therefore lead to a 30% reduction in the overall mortality rate (10.5%).

According to these assumptions, the sample size for an individual randomized trial would be 1730 children. For financial and logistical reasons, we retained an ICC value of 0.005, corresponding to a design effect of 2.16 [31]. Using the estimated mortality of 15% in the control arm, an expected reduction in mortality in the experimental arm of 30%, an alpha of 0.05, a power of 80%, an ICC of 0.005 and 1% of incomplete data, the corresponding sample sizes would be 3780 children. This sample size randomised across the two strategies, in 15 hospitals over 16 periods, resulted in a mean of 15.8 children enrolled per hospital per time period (or 252 children per hospital for the entire study), with no restriction if included numbers exceed this target.

### Data management and confidentiality

Patient data will be recorded into an electronic case report form by study nurses through single data entry on tablets using the REDCap application. All electronic data will be kept on a password-protected, secured server hosted at the University of Bordeaux, accessible only to researchers involved in this trial. Each trial participant will be assigned a unique study identification code.

#### Oversight

The trial is coordinated primarily by the international Central Coordination Unit at University of Bordeaux, France and is overseen by the Trial Steering Committee. A Scientific Advisory Board (SAB) provides advice on the relevance and validity of the project design and implementation, monitors progress and ensures scientific and ethical integrity of the project. An IDMC acts as a consultative board for the SAB and the sponsor. It has access to overall safety and efficacy data, as well as to any information justifying continuation or discontinuation of the trial. However, the IDMC will not apply stopping rules and interim analyses as per standard clinical trials since the stepped wedge design does not allow for it.

### Sub-studies

#### Cost-effectiveness study

We hypothesise that benefits in terms of survival and increased Disability-Adjusted Life Years (DALYs) over a lifetime horizon will justify extra costs incurred by systematic Ultra testing in children with severe pneumonia. A mathematical model will be developed to project health economic outcomes, including TB cases and mortality in children with severe pneumonia. Cost-effectiveness analysis will be from the health payer perspective and only direct healthcare costs will be included. Budget impact analysis will be conducted to evaluate the expected costs of implementing the TB-Speed approach on healthcare budget at 2- and 5-year horizons in the countries participating in the project.

#### Biomarkers studies

Baseline samples, including NPA and stool leftovers, whole blood and plasma samples collected at inclusion, will be frozen and stored at the country clinical trial unit laboratory. The trial provides a unique opportunity to investigate a number of TB biomarkers, which could discriminate active disease from latent TB infection as well as TB from non-TB pneumonia using transcriptomic approaches. It also permits the study of the molecular epidemiology of *Mycobacterium tuberculosis*, and further characterisation of the proteomic, metabolic and immunologic profiles of children presenting with signs of severe pneumonia, with or without TB [32–36]. Biological samples will be retained for 10 years after study completion, unless there are objections expressed by parent(s)/guardian(s).

### Trial status

Recruitment to the trial started in March 2019. Recruitment will continue until September 2020, with the last visit of the last participant in December 2020. The current protocol is Version 2.0 dated November 22nd, 2019.

## Discussion

TB-Speed Pneumonia is a pragmatic diagnostic trial with a stepped-wedge cluster-randomised design, and a stratified randomisation. The stepped wedge cluster-randomised design enables evaluation of the study hypothesis using a pragmatic and operational approach, i.e. for an intervention which remains to be tested in a real-world setting. To our knowledge, this is the first time a stepped wedge trial has been implemented at the international level [37–39].

### Strengths and challenges of the stepped-wedge design

The stepped wedge design is particularly relevant where it is predicted that the intervention will ultimately do more good than harm, as is the case for our intervention, but where there is uncertainty as to its effectiveness and its safety in a specific setting and population [40]. The choice of the stepped wedge design was also based on our hypothesis that the intervention would raise TB awareness among clinicians and may lead to more empirical TB treatment initiated in the intervention arm as compared to the control arm, thus benefitting all children hospitalized, and impacting beyond the research settings. Since clusters are expected to remain until the study ends, they will all eventually implement the intervention. The stepped-wedge design is therefore particularly adapted to capture such effects. From a logistical point of view, a phased roll-out of the intervention is easier to implement in the context of an international multicentre study. Randomisation per hospital is also advantageous due to the difficulty at health facility level of randomising children individually to one of the two strategies, additionally minimising contamination between the two arms.

A major limitation of stepped wedge designs is that blinding of the study team to the intervention is not possible. However, since the primary trial endpoint (mortality at 12 weeks) is not subjective, there is no risk of ascertainment bias. As the intervention relates to the management of children upon hospital admission, outcomes are estimated only from individuals with no prior exposure to the control, thus avoiding carryover (residual) effect [41]. Moreover, in order to guarantee that children benefit from the same quality of care across study sites, adherence to WHO SOC for severe pneumonia will be monitored throughout the study implementation.

Challenges to this design include the need for repeated training activities and increasing workload for coordination teams as more clusters start the intervention. Although all hospitals will finally implement the intervention, some of them will remain in the control arm for a long time, requiring continuous engagement to avoid drop-out and demotivation. In this study, the number of participants is not capped, which can be challenging for resource planning. Since all study sites are expected to start on the same day, as per the stepped wedge design, any delay in opening a site impacts all others.

In this multi-country study with different epidemiological and environmental backgrounds, one can expect to face variability in the number of recruitments across clusters and time-periods (seasonal effect). Although the impact of unequal cluster size on statistical precision has been previously investigated, further research is needed to apply previous results to binary outcomes and to consider size variation across clusters and time-periods [42].

## Conclusion

TB-Speed Pneumonia will test an innovative intervention to diagnose TB in children with severe pneumonia, in a pragmatic cluster randomised stepped-wedge trial. In addition to testing the main hypothesis that molecular detection and early treatment will reduce TB mortality in children, the strength of such pragmatic research is that it helps to provide some evidence regarding the feasibility of the intervention as part of routine care. A systematic review, commissioned by WHO in 2019, provided additional evidence on the use of Xpert MTB/RIF and Ultra as initial diagnostic tests for TB in children, specifically in NPA and stool specimens. The sensitivity of Xpert MTB/RIF (as compared to liquid culture of a respiratory specimen) was of 46 and 61% on NPA and stool specimens respectively, as compared to 65% on sputum and 73% on gastric specimens, and the sensitivity of Ultra was of 46 and 73% on NPA and sputum, respectively. The specificity for all samples was above 98% (97% for Ultra) [43].

Should this intervention be successful, safe and well tolerated, it could be systematically implemented in order to increase TB diagnosis in children with severe pneumonia and reduce childhood mortality due to TB.

## Data Availability

The datasets generated and/or analysed during the current study will be available from the corresponding author on reasonable request.

## Abbreviations

TB: Tuberculosis
WHO: World Health Organization
SOC: Standard of care
Ultra: Xpert MTB/RIF Ultra
NPA: Nasopharyngeal aspirate
CXR: Chest X-ray
Inserm: French National Institute for Health and Medical Research

## References

[1] World Health Organization. Global tuberculosis report 2019. Geneva: World Health Organization; 2019.

[2] Dodd PJ, Yuen CM, Sismanidis C, Seddon JA, Jenkins HE (2017). The global burden of tuberculosis mortality in children: a mathematical modelling study. Lancet Glob Health.

[3] Perez-Velez CM, Marais BJ (2012). Tuberculosis in children. N Engl J Med.

[4] Walker CLF, Rudan I, Liu L, Nair H, Theodoratou E, Bhutta ZA (2013). Global burden of childhood pneumonia and diarrhoea. Lancet Lond Engl.

[5] World Health Organization, Department of Maternal N Child and Adolescent Health, World Health Organization (2014). Revised WHO classification and treatment of pneumonia in children at health facilities: evidence summaries.

[6] Ngari MM, Fegan G, Mwangome MK, Ngama MJ, Mturi N, Scott JAG (2017). Mortality after inpatient treatment for severe pneumonia in children: a cohort study. Paediatr Perinat Epidemiol.

[7] Lazzerini M, Seward N, Lufesi N, Banda R, Sinyeka S, Masache G (2016). Mortality and its risk factors in Malawian children admitted to hospital with clinical pneumonia, 2001-12: a retrospective observational study. Lancet Glob Health.

[8] Sutcliffe CG, Thea DM, Seidenberg P, Chipeta J, Mwananyanda L, Somwe SW (2016). A clinical guidance tool to improve the care of children hospitalized with severe pneumonia in Lusaka, Zambia. BMC Pediatr.

[9] Liu L, Oza S, Hogan D, Chu Y, Perin J, Zhu J (2016). Global, regional, and national causes of under-5 mortality in 2000–15: an updated systematic analysis with implications for the sustainable development goals. Lancet.

[10] Oliwa JN, Karumbi JM, Marais BJ, Madhi SA, Graham SM (2015). Tuberculosis as a cause or comorbidity of childhood pneumonia in tuberculosis-endemic areas: a systematic review. Lancet Respir Med.

[11] Nantongo JM, Wobudeya E, Mupere E, Joloba M, Ssengooba W, Kisembo HN (2013). High incidence of pulmonary tuberculosis in children admitted with severe pneumonia in Uganda. BMC Pediatr.

[12] Chisti MJ, Graham SM, Duke T, Ahmed T, Faruque ASG, Ashraf H (2014). Post-Discharge Mortality in Children with Severe Malnutrition and Pneumonia in Bangladesh. Esposito S, editor. Plos One.

[13] Enarson PM, Gie RP, Enarson DA, Mwansambo C, Graham SM (2010). Impact of HIV on standard case management for severe pneumonia in children. Expert Rev Respir Med.

[14] World Health Organization. Pocket book of hospital care for children: guidelines for the management of common illnesses with limited resources. 2nd edition. Geneva: World Health Organization; 2005.

[15] World Health Organization. Policy statement: automated real-time nucleic acid amplification technology for rapid and simultaneous detection of tuberculosis and rifampicin resistance: Xpert MTB/RIF system. Geneva: World Health Organization; 2011. 26158191

[16] Detjen AK, DiNardo AR, Leyden J, Steingart KR, Menzies D, Schiller I (2015). Xpert MTB/RIF assay for the diagnosis of pulmonary tuberculosis in children: a systematic review and meta-analysis. Lancet Respir Med.

[17] Chisti MJ, Graham SM, Duke T, Ahmed T, Ashraf H, Faruque ASG (2014). A Prospective Study of the Prevalence of Tuberculosis and Bacteraemia in Bangladeshi Children with Severe Malnutrition and Pneumonia Including an Evaluation of Xpert MTB/RIF Assay. Nicol MP, editor. Plos One.

[18] Dorman SE, Schumacher SG, Alland D, Nabeta P, Armstrong DT, King B (2018). Xpert MTB/RIF ultra for detection of mycobacterium tuberculosis and rifampicin resistance: a prospective multicentre diagnostic accuracy study. Lancet Infect Dis.

[19] FIND. Report for WHO. A multicentre non-inferiority diagnostic accuracy study of the Ultra assay compared to the Xpert MTB/RIF assay. Geneva: FIND; 2017.

[20] Marcy O, Ung V, Goyet S, Borand L, Msellati P, Tejiokem M (2016). Performance of Xpert MTB/RIF and alternative specimen collection methods for the diagnosis of tuberculosis in HIV-infected children. Clin Infect Dis.

[21] Zar HJ, Workman L, Isaacs W, Munro J, Black F, Eley B (2012). Rapid molecular diagnosis of pulmonary tuberculosis in children using nasopharyngeal specimens. Clin Infect Dis.

[22] Walters E, Gie RP, Hesseling AC, Friedrich SO, Diacon AH, Gie RP (2012). Rapid diagnosis of pediatric Intrathoracic tuberculosis from stool samples using the Xpert MTB/RIF assay: a pilot study. Pediatr Infect Dis J.

[23] Dim B, Tran T, Borand L. Feasibility, safety and tolerability of nasopharyngeal aspirates and string tests for diagnosis of TB with Xpert® MTB/RIF in HIVinfected children: ANRS 12229 PAANTHER 01 study. Guadalajara: 48th Union World Conference on Lung Health; 2017.

[24] Nicol MP, Spiers K, Workman L, Isaacs W, Munro J, Black F (2013). Xpert MTB/RIF testing of stool samples for the diagnosis of pulmonary tuberculosis in children. Clin Infect Dis Off Publ Infect Dis Soc Am.

[25] Planting NS, Visser GL, Nicol MP, Workman L, Isaacs W, Zar HJ (2014). Safety and efficacy of induced sputum in young children hospitalised with suspected pulmonary tuberculosis. Int J Tuberc Lung Dis Off J Int Union Tuberc Lung Dis.

[26] Song et al. Diagnostic yield of Xpert® MTB/RIF assay and *Mycobacterium tuberculosis* culture on respiratory and non-respiratory specimens among Kenyan children. Cape Town: 46th Union Conference on World Health; 2015.

[27] U.S. Department of Health and Human Services, National Institutes of Health, National Institute of Allergy and Infectious Diseases, Division of AIDS. Division of AIDS (DAIDS) Table for Grading the Severity of Adult and Pediatric Adverse Events, Corrected Version 2.1. 2017 p. 35.

[28] Hemming K, Taljaard M, Forbes A (2017). Analysis of cluster randomised stepped wedge trials with repeated cross-sectional samples. Trials..

[29] Hussey MA, Hughes JP (2007). Design and analysis of stepped wedge cluster randomized trials. Contemp Clin Trials.

[30] Hemming K, Taljaard M, Grimshaw J (2019). Introducing the new CONSORT extension for stepped-wedge cluster randomised trials. Trials..

[31] Hemming K, Taljaard M (2016). Sample size calculations for stepped wedge and cluster randomised trials: a unified approach. J Clin Epidemiol.

[32] Verhagen LM, Zomer A, Maes M, Villalba JA, del Nogal B, Eleveld M (2013). A predictive signature gene set for discriminating active from latent tuberculosis in Warao Amerindian children. BMC Genomics.

[33] Anderson ST, Kaforou M, Brent AJ, Wright VJ, Banwell CM, Chagaluka G (2014). Diagnosis of childhood tuberculosis and host RNA expression in Africa. N Engl J Med.

[34] Zhou M, Yu G, Yang X, Zhu C, Zhang Z, Zhan X (2016). Circulating microRNAs as biomarkers for the early diagnosis of childhood tuberculosis infection. Mol Med Rep.

[35] Walzl G, McNerney R, du Plessis N, Bates M, McHugh TD, Chegou NN (2018). Tuberculosis: advances and challenges in development of new diagnostics and biomarkers. Lancet Infect Dis.

[36] Gjøen JE, Jenum S, Sivakumaran D, Mukherjee A, Macaden R, Kabra SK (2017). Novel transcriptional signatures for sputum-independent diagnostics of tuberculosis in children. Sci Rep.

[37] Shah L, Rojas M, Mori O, Zamudio C, Kaufman JS, Otero L (2015). Implementation of a stepped-wedge cluster randomized design in routine public health practice: design and application for a tuberculosis (TB) household contact study in a high burden area of Lima. Peru BMC Public Health.

[38] Durovni B, Saraceni V, van den Hof S, Trajman A, Cordeiro-Santos M, Cavalcante S (2014). Impact of replacing smear microscopy with Xpert MTB/RIF for diagnosing tuberculosis in Brazil: a stepped-wedge cluster-randomized trial. Plos Med.

[39] Durovni B, Saraceni V, Moulton LH, Pacheco AG, Cavalcante SC, King BS (2013). Effect of improved tuberculosis screening and isoniazid preventive therapy on incidence of tuberculosis and death in patients with HIV in clinics in Rio de Janeiro, Brazil: a stepped wedge, cluster-randomised trial. Lancet Infect Dis.

[40] Hargreaves JR, Copas AJ, Beard E, Osrin D, Lewis JJ, Davey C (2015). Five questions to consider before conducting a stepped wedge trial. Trials..

[41] Copas AJ, Lewis JJ, Thompson JA, Davey C, Baio G, Hargreaves JR (2015). Designing a stepped wedge trial: three main designs, carry-over effects and randomisation approaches. Trials..

[42] Martin JT, Hemming K, Girling A (2019). The impact of varying cluster size in cross-sectional stepped-wedge cluster randomised trials. BMC Med Res Methodol.

[43] World Health Organization. WHO consolidated guidelines on tuberculosis. Module 3: diagnosis – rapid diagnostics for tuberculosis detection. Geneva: World Health Organization; 2020. 33999549

